# Systemic amyloidosis journey from diagnosis to outcomes: a twelve-year real-world experience of a single center in a middle-income country

**DOI:** 10.1186/s13023-022-02584-3

**Published:** 2022-12-05

**Authors:** Roberta Shcolnik Szor, Fabio Fernandes, Angelina Maria Martins Lino, Leonardo Oliveira Mendonça, Fernanda Salles Seguro, Valkercyo Araujo Feitosa, Jussara Bianchi Castelli, Lecticia Barbosa Jorge, Lucas Bassolli de Oliveira Alves, Precil Diego Miranda de Menezes Neves, Evandro de Oliveira Souza, Livia Barreira Cavalcante, Denise Malheiros, Jorge Kalil, Gracia Aparecida Martinez, Vanderson Rocha

**Affiliations:** 1 Serviço de Hematologia, Hemoterapia e Terapia Celular, Hospital das Clínicas and Instituto do Câncer do Estado de São Paulo, Universidade de São Paulo, Av. Dr. Arnaldo, 251, Cerqueira César, São Paulo, SP CEP 01246-000 Brazil; 2 Instituto do Coração (InCor), Universidade de São Paulo, São Paulo, Brazil; 3 Divisão de Neurologia, Hospital das Clínicas, Universidade de São Paulo, São Paulo, Brazil; 4 Departamento de Imunologia Clínica e Alergia, Hospital das Clínicas, Universidade de São Paulo, São Paulo, Brazil; 5 Divisão de Nefrologia, Hospital das Clínicas, Universidade de São Paulo, São Paulo, Brazil; 6 Laboratório de Patologia, Instituto do Coração (InCor), Universidade de São Paulo, São Paulo, Brazil; 7 Grupo Fleury, São Paulo, Brazil; 8 Divisão de Gastroenterologia e Hepatologia Clínica, Hospital das Clínicas, Universidade de São Paulo, São Paulo, Brazil; 9 Divisão de Anatomia Patológica, Hospital das Clínicas, Universidade de São Paulo, São Paulo, Brazil; 10 Department of Hematology, Churchill Hospital, Oxford University, Oxford, UK

**Keywords:** Amyloidosis, Systemic amyloidosis, AL amyloidosis, ATTR amyloidosis, AA amyloidosis

## Abstract

**Background:**

Systemic amyloidosis is caused by the deposition of misfolded protein aggregates in tissues, leading to progressive organ dysfunction and death. Epidemiological studies originate predominantly from high-income countries, with few data from Latin America. Due to the non-specific clinical manifestations, diagnosing amyloidosis is often challenging and patients experience a long journey and delay in diagnosis. This study aimed to assess clinical and laboratory characteristics, the diagnostic journey, and outcomes of patients with biopsy-proven systemic amyloidosis diagnosed between 2009 and 2020 at a university referral center in a middle-income Latin American country. Patients´ medical records were retrospectively reviewed.

**Results:**

One hundred and forty-three patients were included. The median age at diagnosis was 60 years and 54% were male. Until the diagnosis, most of the patients (52%) were seen by at least 3 specialists, the main ones being: general practitioners (57%), nephrologists (45%), and cardiologists (38%). The most common manifestations were renal (54%) and cardiac (41%) disorders, and cachexia was seen in 36% of patients. In 72% of the cases, ≥ 2 biopsies were required until the final diagnosis. The median time from symptoms onset to diagnosis was 10.9 months, and most patients (75%) had ≥ 2 organs involved. The following subtypes were identified: AL (68%), ATTR (13%), AA (8%), AFib (4%), and inconclusive (7%). Median OS was 74.3 months in the non-AL subgroup and 18.5 months in AL. Among AL patients, those with advanced cardiac stage had the worst outcome [median OS 8.6 months versus 52.3 for stage III versus I–II, respectively (*p* < 0.001)]. AL subtype, cardiac involvement, and ECOG ≥ 2 were identified as independent risk factors for reduced survival.

**Conclusions:**

Systemic amyloidosis is still an underdiagnosed condition and the delay in its recognition leads to poor outcomes. Medical education, better diagnostic tools, improvement in access to therapies, and establishment of referral centers may improve patient outcomes in middle-income countries.

**Supplementary Information:**

The online version contains supplementary material available at 10.1186/s13023-022-02584-3.

## Introduction

Systemic amyloidosis (SA) comprises a group of rare diseases caused by tissue deposition of misfolded protein aggregates in the form of insoluble fibrils, leading to progressive organ dysfunction, disability and potentially death [1]. Fourteen proteins have already been described as possible causes of SA, and their precise identification is essential for target therapy, which is specific to each disease subtype [2–7].

Epidemiological data on SA originate mainly from American and European studies. Immunoglobulin light chain (AL) amyloidosis is the most common subtype, with an incidence of 9–12 cases per million per year, and an estimated prevalence of 30,000–45,000 people living with the disease in developed countries [5, 6, 8–13]. The second most recognized subtype is transthyretin amyloid protein (ATTR) amyloidosis, comprising both the variant ATTR (ATTRv) and wild-type ATTR (ATTRwt). While ATTRv´s estimated incidence and prevalence are 0.3 cases per million per year and 5.2 cases per million persons, respectively, ATTRwt amyloidosis has been increasingly recognized, and its estimated prevalence reaches 150 cases per million persons [5, 13–15]. Other less common subtypes of SA include serum amyloid A (AA) amyloidosis, which incidence is decreasing due to the improvement in treatment of underlying chronic inflammatory diseases, and other hereditary and acquired forms of the disease [13, 16].

Amyloidosis affects the heart, kidneys, and peripheral and autonomic nervous system. However, other organs can also be involved, resulting in a wide spectrum of clinical syndromes. Non-specific manifestations are often observed, making the diagnostic process a challenge for physicians, leading patients to experience a long journey characterized by many medical visits, exams, misdiagnosis, and delay in the final diagnosis [17].

Despite recent improvements in diagnosis and treatment of amyloidosis, it remains a morbid condition with elevated early mortality, especially in patients with cardiac dysfunction. Early recognition of the disease and prompt intervention are essential to prevent further organ damage [3, 4, 18, 19].

In Latin America, amyloidosis is still poorly studied, and due to its rarity and underdiagnosis, there are few referral centers. This study aimed to assess clinical and laboratory characteristics of biopsy-proven SA patients, their diagnostic journey, and outcomes at a single university referral center in Brazil. A risk factor analysis for overall survival was performed.

## Methods

### Patients and patient´s journey

We retrospectively reviewed medical records of all patients with biopsy-proven SA diagnosed from 2009 to 2020 at a single public tertiary university referral center, the Hospital das Clínicas da Faculdade de Medicina da Universidade de São Paulo, Brazil. Data were collected on the Research Electronic Data Capture (REDCap) platform [20]. The study was approved by the institution´s Research Ethics Committee. Patients' journey was assessed by the number and types of specialists consulted and the time between symptom onset until diagnosis.

### Classification of the amyloidosis subtype

The etiologic subtype of amyloidosis was confirmed by the identification of the causative protein on tissue biopsies by immunohistochemistry (IHC), indirect immunofluorescence, or mass spectrometry, and by the finding of a pathogenic germline genetic mutation related to amyloidosis. As mass spectrometry was not routinely available, there were cases in which the current diagnostic guidelines could not be followed and typing relied on available data and clinical judgment. Furthermore, in some cases where two or more clinical or laboratory findings suggested different causative proteins, we could not accurately conclude the subtype. These cases were classified as inconclusive.

### Assessment of organ involvement, prognosis, and response to treatment

In AL patients, organ involvement was reported according to the international consensus criteria for AL amyloidosis, and prognosis was assessed by different validated risk models [21–26]. Hematologic and organ responses were reported according to established criteria [27, 28].

### Outcomes

Primary endpoint was overall survival (OS). In the AL subgroup, OS was also assessed according to the standard Mayo Clinic staging and progression to end-stage renal disease was assessed according to renal staging.

### Statistical analysis

Summary statistics were reported in frequencies and median [range or interquartile with 25th and 75th percentiles (Q1-Q3)]. Continuous variables were compared by the Mann–Whitney test. Patients with *inconclusive subtype* were excluded from the comparative analyses between AL versus non-AL subgroups. OS was assessed by the Kaplan–Meier method. Survival data were censored on the date of the last visit or the last contact with the patient, and survival curves were compared using the log-rank test. Univariable risk factor analyses were conducted including the following variables: age, gender, educational level, performance status according to Eastern Cooperative Oncology Group (ECOG), time from symptoms onset to diagnosis, number of involved organs, cardiac and renal involvement, and amyloidosis subtype. A backward multivariable Cox regression was performed to define independent risk factors for OS including all significant variables at a 5% level in univariable analysis. Proportional hazard assumption was verified by Schoenfeld residuals. Hazard ratio and 95% confidence interval (CI) were reported. All statistical tests were two-sided with *p-*values < 0.05 denoting statistical significance. The analyses were performed using R studio version 1.

## Results

One hundred and seventy-one patients were identified with a diagnosis of systemic amyloidosis between 2009 and 2020. Twenty-eight did not have a confirmatory biopsy and were excluded, and 143 patients were eligible for analysis.

### Clinical characteristics, medical history, and patient's journey

Most patients were male (54%; n = 77), median age was 60 years (22–87) and 57% had performance status ≥ 2 according to ECOG scale.

Renal and cardiac disorders were the main clinical presentations (54% and 41%, respectively), and 36% of the patients had cachexia.

Until the diagnosis of amyloidosis was established, most of the patients (52%; n = 74) were seen by 3 or more physicians. After general practitioners (57%), nephrologists and cardiologists were the main specialties consulted (45% and 38%, respectively). A median delay in diagnosis of 10.9 months (0.5–114.5) was observed for the entire cohort. Patients with AL subtype were diagnosed earlier than those with non-AL subtype [9.0 (0.5–90.3) months versus 30.5 (0.9- 108.0), p < 0.001].

### Diagnosis data

In 72% (n = 103) of the cases, two or more biopsies were required for diagnosing amyloidosis. The most common sites of biopsy were bone marrow (57%; n = 81), kidney (42%; n = 60), and fat pad (38%; n = 55). Additional file 1: Table S1 describes other biopsied sites and their positivity for amyloid deposit. More than half of the patients (56%; n = 80) underwent a diagnostic method to identify the precursor protein, namely: indirect immunofluorescence (66%; n = 53), IHC (36%; n = 29) and mass spectrometry (6%; n = 5). Mutations in genes related to amyloidosis were found in 15% (n = 22) of patients, being: transthyretin (TTR) (59%; n = 13); fibrinogen (27%; n = 6); and Mediterranean Fever (MEFV) gene (14%; n = 3). Other diagnostic tools were also performed to investigate the amyloidosis subtype: screening for monoclonal gammopathy (97%, n = 139; FLC unavailable in 55% of them), Tc-PYP scintigraphy in patients with cardiac involvement (18%, n = 19), and assessment of serum amyloid A protein (SAA) levels in patients with suspected AA subtype (67%, n = 8).

The following subtypes of amyloidosis were identified: AL (68%, n = 97), ATTR (13%, n = 19), AA (8%, n = 12), and fibrinogen amyloid protein (AFib) amyloidosis (4%, n = 6). Inconclusive cases comprised 7% (n = 9) of the patients. Table 1 summarizes patients´ general characteristics.

**Table 1:** General characteristics of patients by amyloidosis subtype (n = 143)

Characteristic	Amyloidosis subtype				
	AL	ATTR	AA	AFib	Inconclusive
	n = 97 (%)	n = 19 (%)	n = 12 (%)	n = 6 (%)	n = 9 (%)
Gender					
Male	49 (50.5)	15 (78.9)	4 (33.3)	4 (66.7)	5 (55.5)
Female	48 (49.5)	4(21.1)	8 (66.7)	2 (33.3)	4 (44.4)
Age at diagnosis (years)					
Mean (SD)	60.3 (± 11.3)	59.3 (± 15.2)	46.2 (± 16.4)	57.6 (± 5.8)	61.8 (± 15.3)
Educational level					
Elementary	58 (67.4)	9 (47.4)	6 (54.5)	3 (50.0)	6 (75.0)
Secondary	20 (23.2)	8 (42.1)	3 (27.3)	2 (33.3)	2 (25.0)
University	8 (9.3)	2 (10.5)	2 (18.2)	1 (16.7)	0 (–)
ECOG					
> 2	55 (59.1)	16 (94.1)	3 (30.0)	1 (25.0)	8 (100)
< 2	38 (40.9)	1 (5.9)	7 (70.0)	3 (75.0)	0 (–)
Initial clinical manifestation					
Renal disorders	62 (63.9)	2 (10.5)	5 (50.0)	6 (100)	2 (22.2)
Heart disease	37 (38.1)	12 (63.2)	2 (20.0)	0 (–)	7 (77.7)
Neuropathy	19 (19.6)	12 (63.2)	2 (20.0)	1 (16.7)	1 (11.1)
Gastrointestinal symptoms	23 (23.7)	4(21.1)	5 (50.0)	0 (–)	2 (22.2)
Cachexia	40 (41.2)	6 (31.6)	3 (30.0)	0 (–)	2 (22.2)
Number of specialties consulted until diagnosis					
1–2	43 (44.3)	13 (68.4)	3 (25.0)	4 (66.7)	6 (66.7)
> 3	54 (55.7)	6 (31.6)	9 (75.0)	2 (33.3)	3 (33.3)
Types of specialties consulted until diagnosis					
General practitioner	63 (64.9)	9 (47.4)	5 (41.6)	1 (16.7)	4 (44.4)
Nephrologist	50 (51.5)	2 (10.5)	5 (41.6)	6 (100)	1 (11.1)
Cardiologist	33 (34.0)	11 (57.9)	2 (16.6)	1 (16.7)	8 (88.8)
Neurologist	3 (3.1)	10 (52.6)	4 (33.3)	0 (–)	2 (22.2)
Gastroenterologist	13 (13.4)	3 (15.8)	3 (25.0)	0 (–)	2 (22.2)
Time from symptoms onset to diagnosis (months)					
Median (range)	9.0 (0.5–90.3)	30.6 (1.9–108.0)	40 (3.8–74.7)	10.4 (0.9–36.9)	12.4 (2.8–114.0)

Missing values: educational level (9.0%); ECOG (9.0%); time from symptoms onset to diagnosis (4.0%)

AL = Light Chain Amyloidosis, ATTR = Transthyretin Amyloidosis, AA = Serum Amyloid A Amyloidosis, AFib = Fibrinogen Amyloidosis

### Organ involvement

In most of the patients (75%; n = 107), advanced disease was observed at diagnosis, with ≥ 2 organs involved. Heart and kidney were the main organs affected (75% and 54%, respectively), followed by soft tissue (41%) and autonomic nervous system (24%). Sixteen (11%) patients were on renal replacement therapy. Tables 2 and Additional file 1: Table S2 describe the number and types of organs involved per patient, respectively, according to the amyloidosis subtypes.

**Table 2:** Number of involved organs per patient by amyloidosis subtypes

Number of involved organs	Amyloidosis subtype				
	AL	ATTR	AA	AFib	Inconclusive
	n = 97 (%)	n = 19 (%)	n = 12 (%)	n = 6 (%)	n = 9 (%)
1	10 (10.3)	11 (57.9)	7 (58.3)	6 (100)	2 (22.2)
2	36 (37.1)	6 (31.6)	4 (33.3)	0 (-)	2 (22.2)
> 3	51 (52.6)	2 (10.5)	1 (8.3)	0 (-)	5 (55.5)

AL = Light Chain Amyloidosis, ATTR = Transthyretin Amyloidosis, AA = Serum Amyloid A Amyloidosis, AFib = Fibrinogen Amyloidosis

### Acquired subtypes of amyloidosis

#### AL amyloidosis: clinical and laboratory characteristics, prognostic assessment, and treatment

Among patients with AL amyloidosis, 8% (n = 8) had a previous diagnosis of multiple myeloma. In 5% (n = 5), a small clonal lymphoplasmacytic infiltrate was found in the bone marrow biopsy. Specific signs of AL subtype such as macroglossia and periorbital purpura were present in 17% (n = 16) and 7% (n = 7) of patients, respectively. Lambda light chain was the predominant causative protein (75%; n = 73). Median values of monoclonal protein in serum and urine electrophoresis were 0.9 g/dL (0.2–2.56) and 270 mg/L (10–5520), respectively. Among the 53 patients with available FLC, the median difference between involved and uninvolved FLC was 90.8 mg/L (14.1–3110.6). The median percentage of bone marrow infiltration by plasma cells was 15% (0.8–100%). Fluorescent in situ hybridization analysis was not available in any patient.

Standard Mayo Clinic staging was assessed in 69% (n = 67) of patients. Most of them (66%; n = 44) were stage III, and among them, 55% (n = 24) were stage IIIb according to the European staging of advanced cardiac involvement. Revised Mayo Clinic staging was available in 34% (n = 33) of patients, 58% (n = 19) of them being classified as stages III or IV. Renal staging was evaluated in 97% (n = 94) of patients. A predominance of stages I to II (80%; n = 75) was observed and 16% (n = 15) of the patients were on renal replacement therapy.

Chemotherapy was administered to most patients (84%; n = 81), and the main regimens were alkylating-based (melphalan 48%, n = 39 and cyclophosphamide 47%, n = 38). Thalidomide was used in 19% (n = 15) of cases, and bortezomib in 16% (n = 13). The median number of chemotherapy cycles per patient was 4 (1–13). Autologous stem cell transplantation (ASCT) was performed in 14% (n = 14) of patients, and 93% of them had received prior chemotherapy. Only one patient (1%) underwent kidney transplantation. Exclusive supportive measures were offered to 15% (n = 15) of patients. Among 55 patients with hematological response assessment, 15% (n = 8) achieved complete response, 9% (n = 5) very good partial response, 25% (n = 14) partial response, 25% (n = 14) no response, and 25% (n = 14) had disease progression.

#### Wild-type ATTR amyloidosis and “de novo” ATTR-PN: clinical and laboratory characteristics, and treatment

Two patients were diagnosed with *confirmed* ATTRwt amyloidosis based on endomyocardial and fat pad biopsies showing TTR positive amyloid deposit by IHC and mass spectrometry, respectively, with no TTR mutation. Isolated heart failure was the clinical manifestation in both cases. The first patient was treated with doxycycline and the other with tafamidis.

Two other patients were diagnosed with p*robable* “de novo” ATTR-PN. Both had received a domino liver transplantation from donors with known ATTRv and developed sensorimotor peripheral neuropathy 10 and 13 years after the transplant. Nerve biopsies confirmed amyloid deposition, and genetic sequencing had not been performed. One patient was treated with a second liver transplantation, and the other received supportive treatment.

#### AA amyloidosis: clinical and laboratory characteristics, and treatment

All 12 patients with AA subtype had a probable etiologic diagnosis due to an underlying inflammatory condition, and absence of features of other subtypes. Of the 8 patients with SAA assessment, 75% (n = 6) had elevated levels. Half of the patients presented with kidney involvement, and 42% (n = 5) had amyloid cardiomyopathy. Familial Mediterranean Fever was the underlying condition in 25% (n = 3) of patients with AA amyloidosis. All of them harbored mutations linked to severe phenotypes, in homozygous or compound heterozygous status, in cys or transposition, along the MEFV gene (p.Met694Val and p.Val726Ala). One patient with a monogenic form of AA amyloidosis was successfully treated with interleukin 1 inhibitor, while the others had clinical and laboratory resolution with colchicine. The other patients had the following inflammatory diseases: rheumatoid arthritis (17%; n = 2), non-specified autoinflammatory diseases (17%; n = 2), and immunoglobulin G4-related disease, vasculitis, combined polymyositis/primary biliary cholangitis, TRAPS syndrome, and Sweet syndrome (8%; n = 1 each). One patient was treated with interleukin 6 inhibitor with a laboratory response, another with anti-tumor necrosis factor agents, and the others received different immunosuppressive regimens, such as corticosteroids, cyclophosphamide, azathioprine, mycophenolate, and rituximab.

### Hereditary subtypes of amyloidosis

#### Hereditary/variant ATTR amyloidosis: clinical and laboratory characteristics, and treatment

Thirteen patients had ATTRv, with the following mutations identified: p.Val50Met (54%; n = 7), p.Val142Ile (38%; n = 5), and p.Glu109Lys (8%; n = 1). Among patients with p.Val50Met mutation, the median age at diagnosis was 42 years (30–75). All of them had sensorimotor polyneuropathy, 71% autonomic dysfunction, 57% had ATTR-CM and 14% kidney involvement. Liver transplantation was performed in 43% (n = 3) of the cases and 29% (n = 2) received tafamidis. The other patients received immunosuppressive agents as treatment for a misdiagnosed inflammatory polyneuropathy. Patients carrying p.Val142Ile mutation had a median age of 67 years (54–69) at diagnosis, all of them presented with ATTR-CM, 20% had ATTR-PN, and 40% carpal tunnel syndrome, and the patient with p.Glu109Lys mutation was diagnosed by the age of 45 years and had a mixed phenotype of ATTR-CM and ATTR-PN. They received supportive therapy.

#### AFib amyloidosis: clinical and laboratory characteristics, and treatment

All patients with AFib amyloidosis (n = 6) had a confirmed diagnosis by the presence of amyloid deposit in a kidney biopsy and a p.Glu545Val mutation in the fibrinogen A alpha-chain gene. Kidney was the only organ involved. No patient was on renal replacement therapy at diagnosis. Supportive treatment was performed in 83% (n = 5) of patients, and one (17%) received bortezomib and dexamethasone as AL amyloidosis before confirming AFib. Progression to end-stage renal disease occurred in all patients with an interval from diagnosis ranging from 2 to 94 months.

### Outcomes

**Fig. 1:**
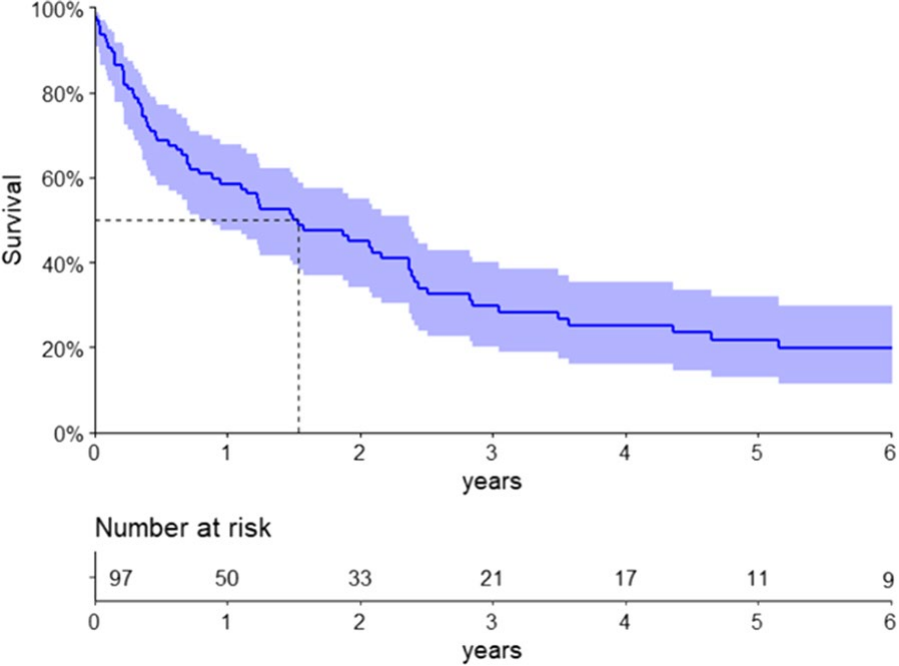
Overall survival of patients with light chain amyloidosis

**Fig. 2:**
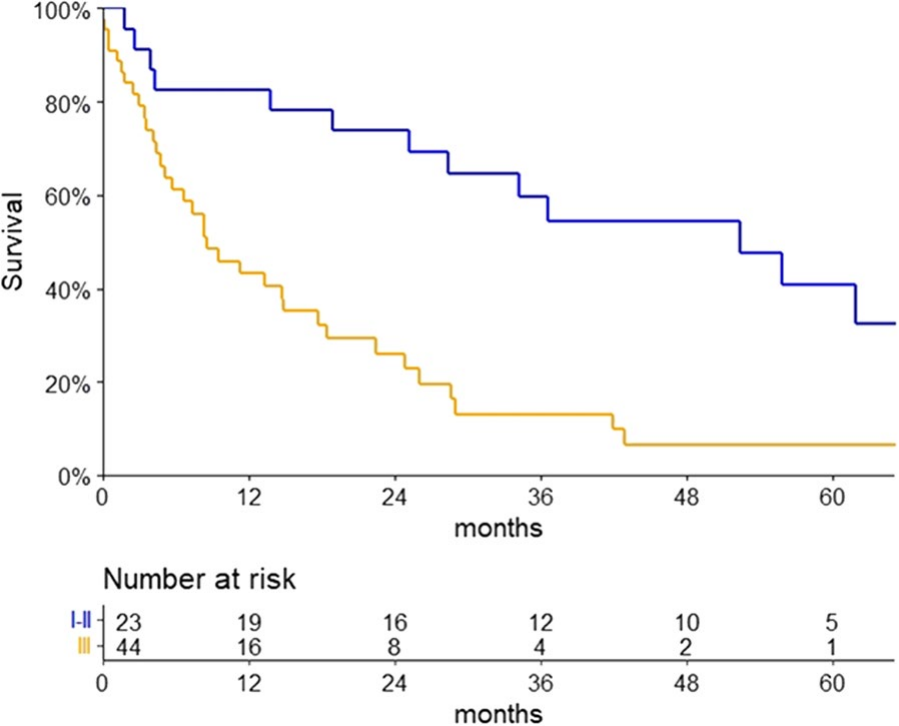
Overall survival of patients with light chain amyloidosis stratified by the standard Mayo Clinic cardiac staging

The median follow-up time was 56.3 months (Q1-Q3 22.6–106.8). In the non-AL subgroup, a median OS of 74.3 months (95% CI 32.6–not reached) was observed. Among patients with AL subtype, the median OS was 18.5 months (95% CI 10.7 – 28.4), as shown in Fig. 1. Patients with cardiac stage III according to the standard Mayo Clinic staging had a decreased OS compared to stages I–II [8.6 (95% CI 4.7–14.8) months versus 52.3 (95% CI 25.2–73.6), respectively, p < 0.001], as shown in Fig. 2.

Early mortality in the first year after the diagnosis of amyloidosis occurred in approximately one third of patients (31%, n = 44), most of them (86%; n = 38) being of the AL subtype.

Regarding renal outcomes in the AL subgroup, 18% (n = 15) of patients progressed to renal replacement therapy. Patients with stage III renal disease had a higher rate of progression to dialysis in 2 years compared to stages I–II [44.1% (95% CI 21.0–76.0) versus 20.6% (95% CI 11.1—36.5), respectively].

As shown in Table 3, we identified ECOG ≥ 2, AL subtype, and cardiac involvement as independent risk factors for decreased OS [HR 1.68 (95% CI 1.04–2.72), HR 2.44 (95% CI 1.26–4.72), and HR 3.27 (95% CI 1.55–6.90), respectively].

**Table 3:** COX regression model to evaluate risk factors associated with overall survival

Variable	Univariable		Multivariable^¥^	
	HR (95%CI)	p value	HR (95%CI)	p value
Age (years)	1.02 (1.0–1.04)	0.029	–	–
Gender (Female vs. Male*)	0.84 (0.55–1.30)	0.442	–	–
Educational level (elementary vs. secondary/university*)	1.68 (1.01–2.79)	0.044	–	–
ECOG (> 2 vs. < 2*)	1.92 (1.21–3.04)	0.006	1.68 (1.04–2.72)	0.035
Time from symptoms onset to diagnosis (months)	0.99 (0.98–1.0)	0.110	–	–
Number of involved organs (> 2 vs. < 2*)	3.54 (1.91–6.56)	< 0.001	–	–
Cardiac involvement (Yes vs. No*)	4.88 (2.48–9.60)	< 0.001	3.27 (1.55–6.90)	0.020
Kidney involvement (Yes vs. No*)	1.01 (0.66–1.55)	0.967		
Subtype (AL vs. Non-AL*)^#^	3.22 (1.77–5.84)	< 0.001	2.44 (1.26–4.72)	0.008

Missing values: educational level (9.0%); ECOG (9.0%)

AL = Light Chain Amyloidosis; CI = Confidence Interval; HR = Hazard Ratio; mo = months; vs. = versus

^*^Reference

^#^Inconclusive subtype excluded (n = 9)

^¥^Multivariable model (n = 122); C-Index = 0.70

## Discussion

Our study shows that diagnosing and treating SA is a challenge in Brazil, with most of the patients being seen by at least 3 specialists with a delay in diagnosis of almost a year. These findings are similar to previous reports in amyloidosis. In a survey with 533 participants, most of the diagnoses were made within one year of initial symptoms (63%), after ≥ 3 physicians consulted (69%) [17]. The delay in diagnosing AL and ATTR subtypes in our cohort is also similar to previously published data: a median time of 7.1 months to diagnose AL amyloidosis was reported in a study of 324 American patients, and in a cohort of 148 Brazilian subjects with ATTRv, a delay of 2.8 and 5.1 years were reported for early and late-onset presentations, respectively [29, 30].

Our findings showed that patients with a non-AL subtype were diagnosed later compared to AL, which may be explained by the direct toxicity of light chains as an additional mechanism of organ damage in AL amyloidosis, leading to a multisystemic presentation with faster deterioration of organ function [6].

Regarding amyloid typing, it was not possible to establish an accurate diagnosis in almost 10% of the patients. This may reflect the unavailability of mass spectrometry and the high frequency of inconclusive results of antibody-based methods, such as IHC and immunofluorescence. The pitfalls related to immune-mediated methods are well known in the literature, and satisfactory results are reported by reference laboratories with expertise in this field, especially when combining commercial and *in-house* antibodies, which are not available in our center [31, 32]. Another factor that may have hindered the identification of the causative protein was the unavailability of some useful diagnostic tools, such as mutational panels, assessment of SAA, and FLC levels in the public health system, during the diagnostic period of the study.

The frequencies of the amyloidosis subtypes observed in our study (AL predominance followed by ATTR, AA, and hereditary non-ATTR) are in agreement with other cohorts from the United States and Europe [13, 17]. In the largest cohort study to date, Ravichandran et al. published epidemiological data on 11,006 patients followed from 1987 to 2019 in the United Kingdom National Amyloidosis Centre and found 55% of AL, 21% ATTR, 8% AA and 2% of hereditary non-ATTR subtypes [13]. Although ATTRwt amyloidosis is becoming increasingly diagnosed, it represented only a few patients in our cohort, which may be due to our eligibility criteria that required a biopsy-proven diagnosis of amyloidosis, excluding patients with ATTRwt identified by the noninvasive algorithm [33]. Regarding ATTRv, previous studies have shown that the main variant of TTR in Brazil is p.Val50Met, although other mutations have also been described, emphasizing the miscegenation of our population [34–40]. Among the 9 patients with ATTRv in our cohort, similar proportions of p.Val50Met and p.Val142Ile mutations were found, and only one patient had p.Glu109Lys. The small number of cases in our study may reflect the exclusion of ATTRv cases diagnosed without the need of a confirmatory biopsy, by the finding of a TTR mutation combined with a typical clinical presentation and/or a positive family history.

Comparing our AL amyloidosis subgroup to the American cohort reported by Schulman et al., our patients had more cardiac involvement (75% versus 50%), and fewer patients received a proteasome inhibitors (16% versus 49%) or underwent ASCT (14% versus 27%) [29]. Although recent publications have shown an improvement in survival of AL amyloidosis associated with therapeutic advances in the last decades, we observed poor outcomes in our AL subgroup. The median OS of 18.5 months is similar to that reported by Ravichandran et al. for patients diagnosed before 2005, in comparison to those diagnosed in the last decade (18 months versus > 5 years, respectively) [13, 41]. Moreover, Schulman et al. showed that patients diagnosed more than 6 months after symptoms onset had a lower probability of survival, with an increase of 2% in the risk of mortality for each month of delay in diagnosis [29]. In our cohort, the median time of 9.0 months to diagnose AL amyloidosis places our patients in this high-risk subgroup. Altogether, the delay in diagnosis, a multi-organ involvement, advanced cardiac stages, and the lack of access to medications may explain our poor outcomes.

The reduced OS of AL patients observed in our cohort reinforces the well-known multisystemic presentation, greater aggressiveness, and faster organic deterioration of AL in comparison to other subtypes. Despite the development of better treatment options and diagnostic tools leading to improvements in survival in the last decades, the limited access to target medications for AL and ATTR further challenge the management of SA and explain our findings.

Finally, Latin American studies on amyloidosis include reports on AL and ATTR from Argentina, Chile, and Mexico, but data on AFib and AA are even more scarce [42–48]. To date, our study is the first to assess the 12-year real-world experience of a Brazilian single public university center in different amyloidosis subtypes. It is also the largest cohort study of patients with AL amyloidosis in Brazil and Latin America. However, some limitations might be considered: as a retrospective cohort study, some data were unavailable in medical records, the number of patients who lost follow-up was relatively high, and precise information on symptom onset date was sometimes unavailable.

## Conclusion

In conclusion, since amyloidosis is a rare disease, with non-specific signs and symptoms, it is underrecognized by many physicians, making it an underdiagnosed condition. This study raises medical awareness of the challenges of diagnosing and treating amyloidosis in Brazil and Latin America, highlighting the need of continuous medical education and the relevance of establishing local and national registries of rare diseases, as well as referral centers with availability of diagnostic tools and specific treatments. Altogether, these measures may improve patient care, aiming to achieve better outcomes in the future.

## Supplementary Information

**Additional file 1.** **Table S1.** Frequency of biopsied sites and positivity rates for amyloid deposit. **Table S2.** Organic involvement by amyloidosis subtypes.

## Data Availability

All data were collected and stored on the Research Electronic Data Capture (REDCap) platform. The datasets used and analyzed during the current study are available from the corresponding author on reasonable request.

## Abbreviations

SA: Systemic Amyloidosis
AL: Immunoglobulin Light Chain
ATTR: Transthyretin amyloid protein
ATTRv: Variant transthyretin amyloid protein
ATTRwt: Wild type transthyretin amyloid protein
AA: Serum amyloid A
IHC: Immunohistochemistry
FLC: Free light chain
ATTR-CM: Transthyretin amyloid cardiomyopathy
Tc-PYP: Technetium-pyrophosphate
ATTR-PN: Transthyretin amyloid polyneuropathy
OS: Overall survival
ECOG: Eastern Cooperative Oncology Group
CI: Confidence interval
TTR: Transthyretin
MEFV: Mediterranean Fever
SAA: Serum amyloid A protein
AFib: Fibrinogen amyloid protein
ASCT: Autologous stem cell transplantation
HR: Hazard ratio
