# Supplementary material for: Systemic amyloidosis journey from diagnosis to outcomes: a twelve-year real-world experience of a single center in a middle-income country
Source: Orphanet J Rare Dis. 2022 Dec 5;17:425. doi: 10.1186/s13023-022-02584-3 (PMC9724300; doi:10.1186/s13023-022-02584-3)
Supplement: Supplementary file 1 — Additional file 1. Table S1. Frequency of biopsied sites and positivity rates for amyloid deposit. Table S2. Organic involvement by amyloidosis subtypes. [file 13023_2022_2584_MOESM1_ESM.pdf]

Supplementary Appendix

Table of contents

Table S1. Frequency of biopsied sites and positivity rates for amyloid deposit.

Table S2. Organic involvement by amyloidosis subtypes

Table S1. Frequency of biopsied sites and positivity rates for amyloid deposit.

Biopsy sites	Number of biopsies (%)	Positivity (%)
Bone Marrow	81 (56.6)	11 (13.5)
Kidney	60 (41.9)	58 (96.6)
Abdominal Fat	55 (38.4)	27 (49.0)
Gastrointestinal Tract	25 (17.4)	10 (40.0)
Heart	21 (14.6)	21 (100)
Skin	18 (12.5)	9 (50.0)
Lymph node	8 (5.5)	6 (75.0)
Nerve	7 (4.8)	5 (71.4)
Sallivary Gland	6 (4.1)	4 (66.6)
Lung / Pleural	6 (4.1)	5 (83.3)
Liver	5 (3.4)	4 (80.0)
Muscle	3 (2.0)	3 (100)
Gingival	3 (2.0)	2 (66.6)
Bladder	2 (1.3)	1 (50.0)
Tongue	1 (0.6)	1 (100)
Spleen	1 (0.6)	1 (100)

Table S2. Organic involvement by amyloidosis subtypes

Involved Organ	Amyloidosis Subtype				
	AL<br>n = 97 (%)	ATTR<br>n = 19 (%)	AA<br>n = 12 (%)	AFib<br>n = 6 (%)	Inconclusive<br>n = 9 (%)
Heart	80 (82.5)	14 (73.7)	5 (41.7)	0 (-)	8 (88.9)
Kidney	61 (62.9)	1 (5.3)	6 (50.0)	6 (100)	3 (33.3)
Soft Tissue	47 (48.5)	3 (15.8)	4 (33.3)	0 (-)	4 (44.4)
Autonomic Peripheral Nervous System	22 (22.7)	8 (42.1)	1 (8.3)	0 (-)	3 (33.3)
Somatic Peripheral Nervous System	17 (17.5)	11 (57.9)	0 (-)	0 (-)	2 (22.2)
Liver	19 (19.6)	0 (-)	1 (8.3)	0 (-)	2 (22.2)
Gastrointestinal Tract	11 (11.3)	0 (-)	1 (8.3)	0 (-)	0 (-)
Respiratory Tract	6 (6.2)	0 (-)	0 (-)	0 (-)	1 (11.1)

AL = Light Chain Amyloidosis, ATTR = Transthyretin Amyloidosis, AA = Serum Amyloid A Amyloidosis, AFib = Fibrinogen Amyloidosis
